# Effects of the Light/Dark Phase and Constant Light on Spatial Working Memory and Spine Plasticity in the Mouse Hippocampus

**DOI:** 10.3390/cells12131758

**Published:** 2023-06-30

**Authors:** Jane K. Schröder, Laila Abdel-Hafiz, Amira A. H. Ali, Teresa C. Cousin, Johanna Hallenberger, Filipe Rodrigues Almeida, Max Anstötz, Maximilian Lenz, Andreas Vlachos, Charlotte von Gall, Federica Tundo-Lavalle

**Affiliations:** 1 Institute of Anatomy II, Medical Faculty, Heinrich-Heine-University, Universitätsstraße 1, 40225 Düsseldorf, Germany; jane.schroeder@ukbonn.de (J.K.S.); laila.abdel-hafiz@med.uni-duesseldorf.de (L.A.-H.); amira.ali@med.uni-duesseldorf.de (A.A.H.A.); tecou100@uni-duesseldorf.de (T.C.C.); johanna.hallenberger@med.uni-duesseldorf.de (J.H.); filipe.rodriguesalmeida@med.uni-duesseldorf.de (F.R.A.); max.anstoetz@med.uni-duesseldorf.de (M.A.); federica.tundo-lavalle@med.uni-duesseldorf.de (F.T.-L.); 2 Department of Pediatric Hematology and Oncology, Medical Faculty, University of Bonn, Venusberg-Campus 1, 53127 Bonn, Germany; 3 Department of Human Anatomy and Embryology, Faculty of Medicine, Mansoura University, El-Gomhoria St. 1, Mansoura 35516, Egypt; 4 Institute of Neuroanatomy and Cell Biology, Hannover Medical School, Carl-Neuberg-Straße 1, 30625 Hannover, Germany; lenz.maximilian@mh-hannover.de; 5 Department of Neuroanatomy, Institute of Anatomy and Cell Biology, Faculty of Medicine, University of Freiburg, 79104 Freiburg, Germany; andreas.vlachos@anat.uni-freiburg.de

**Keywords:** circadian, diurnal, synaptopodin, constant darkness, synapse, hippocampus, GluR1 (GluA1), corticosterone

## Abstract

Circadian rhythms in behavior and physiology such as rest/activity and hormones are driven by an internal clock and persist in the absence of rhythmic environmental cues. However, the period and phase of the internal clock are entrained by the environmental light/dark cycle. Consequently, aberrant lighting conditions, which are increasing in modern society, have a strong impact on rhythmic body and brain functions. Mice were exposed to three different lighting conditions, 12 h light/12 h dark cycle (LD), constant darkness (DD), and constant light (LL), to study the effects of the light/dark cycle and aberrant lighting on the hippocampus, a critical structure for temporal and spatial memory formation and navigation. Locomotor activity and plasma corticosterone levels were analyzed as readouts for circadian rhythms. Spatial working memory via Y-maze, spine morphology of Golgi–Cox-stained hippocampi, and plasticity of excitatory synapses, measured by number and size of synaptopodin and GluR1-immunreactive clusters, were analyzed. Our results indicate that the light/dark cycle drives diurnal differences in synaptic plasticity in hippocampus. Moreover, spatial working memory, spine density, and size and number of synaptopodin and GluR1 clusters were reduced in LL, while corticosterone levels were increased. This indicates that acute constant light affects hippocampal function and synaptic plasticity.

## 1. Introduction

Rhythms in behavior and physiology such as rest/activity and hormone secretion, which are driven by an internal clock, persist even in the absence of rhythmic environmental cues with a period length of about 24 h (i.e., circadian) [1]. The master circadian clock resides in the hypothalamic suprachiasmatic nucleus (SCN) that controls subordinate circadian clocks in the brain and the body via neuronal connections such as the autonomic nervous system and hormones such as melatonin and glucocorticoids [2]. In particular, glucocorticoids, which play an important role in glucose homeostasis, provide a rhythmic signal for entraining subordinate circadian oscillators [3]. Glucocorticoids are also key regulators in physiological and behavioral response to stress. At the cellular level, circadian rhythms in gene expression are driven by a molecular clockwork composed of autoregulatory feedback loops of clock genes [2]. The phase and period of circadian rhythms are entrained by rhythmic environmental stimuli “Zeitgeber”, of which the light/dark cycle is the most important [1]. Moreover, in nocturnal animals such as laboratory mice, bright light is highly aversive and suppresses locomotor activity (masking) [4]. Although circadian photoentrainment and masking are two separate mechanisms, they work in a complementary way in nature [5]. Rods and cones, as well as intrinsically photosensitive ganglion cells (ipRGCs) in the retina, receive the light information and provide the light input not only for image formation via the visual system but also for non-image-forming light responses such as circadian photoentrainment and the pupillary light reflex [6]. The ipRGCs directly project not only to the SCN but also to other regions in the hypothalamus such as the ventral preoptic area and lateral hypothalamus controlling rhythms in rest/activity, heart rate, and glucocorticoids, other diencephalic regions such as habenular nuclei, and basal forebrain regions such as nucleus accumbens and medial amygdala, which provide emotional components for cognitive functions [7,8,9]. Humans living in modern society experience much lower light intensity (400–600 lx) during the day compared to sunlight (~100,000 lx) and a higher illumination of 100–300 lx in the evening/night compared to moonlight (0.1–0.3 lx) due to the lighting conditions indoors and street illumination [9,10]. Furthermore, light-emitting devices such as TVs, computers, tablets, and smartphones, providing 30–50 lx of light, are increasingly used at night and affect circadian timing and performance [9,11]. In particular, chronic exposure to light at night can result in a disruption of circadian rhythms with potential negative consequences for health in general and mental health in particular [10]. Furthermore, sleep and behavior of mice are highly sensitive to light [12]. In the laboratory, exposure of nocturnal rodents to constant light (LL) leads to a gradually increasing disruption of circadian rhythms such as rhythms in serum corticosterone levels [13] and locomotor activity [14]. However, little is known about the role of the circadian system and the effect of light on structural and functional plasticity of the hippocampus, the key structure in temporal and spatial learning and memory that enables navigation.

Spatial working memory is correlated with hippocampal, particularly the dorsal part, structural synaptic plasticity such as formation, elimination, and morphological changes of dendritic spines [15], i.e., dendritic protrusions containing the postsynaptic part of excitatory synapses [16]. Changes in spine morphology are closely related to functional synaptic plasticity such as long-term potentiation (LTP), a long-lasting strengthening of glutamatergic synapses based on recent patterns of activity. Hippocampal LTP depends on calcium transients mediated by, e.g., NMDA receptors or voltage-gated calcium channels. Moreover, the strength of excitatory synapses is modulated in a homeostatic manner aimed at stabilizing neuronal function upon perturbations in network activity. Consistently, synaptic strength increases in response to persisting reduction in neuronal activity or reduction in the number of synapses, e.g., after denervation [17,18]. This compensatory adjustment is achieved, at least in part, through changes in the GluR1 subunit of postsynaptic AMPA glutamate receptors (GluR) [19,20,21], presumably via calcium-dependent negative feedback mechanisms [19,22]. Synaptic insertion of GluR1 fulfills two functions, increased synaptic strength and structural stabilization through increased spine size [23].

Synaptopodin is an actin-associated protein and an essential component of the spine apparatus, which is important for the regulation of postsynaptic calcium concentration and for the actin spinoskeleton in mature spines [24,25,26]. Synaptopodin is associated with structural synaptic plasticity, such as motility, stability, and long-term survival of spines [27,28,29,30], and is implicated in homeostatic synaptic plasticity [17,18]. The presence of synaptopodin in spines is associated with larger responses to glutamate and enhanced prevalence of GluR1 in dendritic spines [26,31,32]. Mice with targeted deletion of synaptopodin exhibit a loss in spine apparatus, reduced hippocampal LTP, and spatial learning deficits [25], and they lack the compensatory increase in synaptic strength in response to denervation [17]. Importantly, synaptopodin cluster size corresponds to a homeostatic increase in synaptic strength, e.g., in response to reduced neuronal network activity [17].

There is increasing evidence that the circadian system modulates synaptic plasticity. The density of spines [33] and the number of synapses [34] vary throughout the day in the hippocampus and the somatosensory cortex, respectively. Moreover, multiple genes and proteins associated with synaptic plasticity, including CREB1 and HDAC, show a time-of-day-dependent expression in the hippocampus [35]. Importantly, time-of-day-dependent changes in the number of excitatory synapses located on dendritic spines in the somatosensory cortex may be light-induced as they vanish in constant dark conditions [34].

In this study, we investigated the role of the light/dark cycle and the effect of acute constant light on hippocampal function and structure in mice. The mice were kept under three distinct lighting conditions: standard condition of 12 h light/12 h darkness (LD), acute constant darkness (DD) to unmask circadian rhythms, and acute constant light (LL). Y-maze was performed in the (former) light and dark phases to test hippocampus-dependent spatial working memory. In addition, mice were sacrificed in (former) light and dark phases to analyze spine morphology, and the number and size of immunolabeled synaptopodin and GluR1 clusters in the hippocampus. We found that day/night differences in the performance in the Y-maze and in number and size of synaptopodin clusters were abolished under constant dark conditions although locomotor activity is still rhythmic. Moreover, constant light results in a significant reduction in performance in the Y-maze and in the number and size of synaptopodin clusters, as well as an increase in plasma corticosterone levels. Thus, our data suggest that the light/dark cycle, rather than rest/locomotor activity, has an enhancing effect, while constant light has a deleterious effect on functional and structural hippocampal plasticity.

## 2. Materials and Methods

### 2.1. Experimental Animals

Male C57Bl/6J mice (8–12 weeks old) were obtained from Janvier Labs (Le Genest-Saint-Isle, France) and housed in single standard cages in light- and soundproof cabinets with automatic time switch (Beastmaster, Mannheim, Germany). The light intensity during the light phase was 400 lx. All mice had free access to food and water ad libitum. The mice were randomly assigned to three groups, which were kept under different lighting conditions. Group 1 was kept under a standard condition of 12 h light and 12 h darkness (LD) [light on at 6:00 a.m. = zeitgeber time (ZT) 00] for 16 days. Group 2 was kept under LD for 14 days, followed by at least 38 h in constant darkness (DD) [circadian time (CT) 00 = lights on in the former light phase] before being sacrificed. Group 3 was kept under LD for 14 days, followed by at least 38 h in constant light (LL) [disrupted time (DT) 00 = lights on in the former light phase] before being sacrificed. For immunofluorescence and serum analyses, mice were sacrificed every 4 h, for a total of six timepoints, starting 2 h after lights on for LD animals, 2 h after former lights on for DD, and 2 h after former lights off for LL (ZT/CT/DT 02, 06, 10, 14, 18, and 22). Mice were kept in at least 38 h of constant darkness/constant light. For Golgi staining, mice were kept as described above and sacrificed at two different timepoints: 2 h after lights/former lights on and 2 h after lights/former lights off. All animal experiments were approved by the local government, North Rhine-Westphalia State Agency for Nature, Environment, and Consumer Protection, Germany (Approval number: 84-02.04.2013.A358) and in agreement with the international guidelines on the ethical use of animals [36]. All efforts were exerted to decrease the number and suffering of animals.

### 2.2. Analysis of Spontaneous Locomotor Activity

The spontaneous locomotor activity of all mice was recorded for 16 days using on-cage infrared movement detectors linked to a monitoring system (Mouse-E-Motion, infra-e-motion, Hamburg, Germany) and analyzed using Clocklab software (R2014a (8.3.0.532), Actimetrics, Wilmette, IL, USA). During the three different lighting conditions, the percentage of activity counts during the light or former light of the total activity was estimated and the relative power of the 24 h period was calculated by fast Fourier transformation.

### 2.3. Corticosterone Assay

For the corticosterone assay, blood was drawn from the right atrium, collected in EDTA sample tubes, and centrifuged at 4 °C for 15 min at 1400× *g*. Then, 25 µL of the blood plasma was subjected to a corticosterone ELISA Kit 2018 (assay sensitivity 2 to 22 pg/mL ab108821, Abcam, Cambridge, UK) according to the manufacturer’s protocol. Optical density was analyzed using a plate reader (Multiskan FC, ThermoScientific, Waltham, MA, USA) at 450 nm. Corticosterone concentration was calculated according to the standard curve.

### 2.4. Exploration Activity and Hippocampus-Dependent Spatial Working Memory

To assess hippocampus-dependent spatial working memory, mice were subjected to a spontaneous spatial alternation behavior task using the Y-maze. This test utilizes the congenital tendency of mice to explore novelty. The mice frequently visit the relatively novel places that were not visited quite recently when freely allowed to choose among respective alternatives in the Y-maze. This alternation behavior is provoked by spatial novelty, thus requiring the integrity of basic spatial working memory, and is sensitive to hippocampal lesions [37,38]. The maze (length: 35 cm; width: 5.3 cm; height: 8 cm) had black plexiglass walls and an open roof. Three equally spaced arms (4.5 cm wide, 30 cm long, and 15 cm height each arm), labeled A, B, and C, were arranged radially from a triangle-shaped central platform. The Y-maze was surrounded by extra-maze visual cues, e.g., posters. In light phase testing, diffuse illumination by LED lights provided a light density of about 112 lx in the center platform and 97 lx in each of the three arms. In dark phase testing, infrared LED lights illuminated the Y-maze. A camera Gig E monochrome with infrared sensor, mounted 60 cm above the maze, linked to a computer-based tracking software system (Ethovision XT 8, Noldus, Wageningen, The Netherlands), was used for continuous tracking of the mice. Mice were habituated to the experimenter’s handling before testing. At the beginning of the experiment, each animal was placed on the central platform and allowed to freely explore the maze for a total trial duration of 7 min. Successful entry into an arm was scored when the animal entered it with all four paws. The number and sequence of arm entries was manually recorded. Between sessions, the apparatus was cleaned with 70% ethanol to eliminate odor cues. The following parameters were considered: (a) total number of entries, as a measure for exploration activity; (b) correct alternations, reflected by the number of triplets/number of triads containing consecutive entries into all three arms without reentries into an arm entered during the last two entries (i.e., CAB, ABC, ACB, etc.); (c) spontaneous alternation, calculated as the number of correct alternations/(total number of entries − 2)  ×  100%, thus reflecting spatial working memory corrected by the exploration activity. Care was taken to ensure that the mice were stress-free during testing. Experiments were conducted in the light/former light and dark/former dark phases by an investigator that was blinded to the experimental conditions.

### 2.5. Immunofluorescence and Quantitative Analysis

Mice were deeply anesthetized using ketamine/xylazine (100 mg/10 mg/kg body weight, respectively) and then transcardially perfused with 0.9% NaCl followed by 4% paraformaldehyde using a Ministar Peristaltic Pump (World Precision Instruments, Sarasota, FL, USA). Brains were removed from the skull and post-fixed for 24 h in 4% formalin. Brains were cut using a Vibratome (VT 1200S, Leica, Bensheim, Germany) into 50 µm thick free-floating sections. Coronal sections at Bregma −1.70 mm containing the dorsal hippocampus were selected for dorsal hippocampus staining [39]. Brain sections of all experimental groups were stained simultaneously to avoid variability between staining sessions. Brain sections were incubated for 1 h with 10% normal goat serum (NGS) in PBS containing 0.5% Triton X-100, to reduce unspecific binding of the secondary antibody, and subsequently incubated for 48 h at 4 °C with rabbit anti-synaptopodin (1:1000 SE-19, Sigma Aldrich, St. Louis, MO, USA) or with rabbit anti-GluR1 (1:1000, Cat. #05-855R, Millipore, Burlington, MA, USA) in PBS, 10% NGS, and 0.1% Triton X-100. After washing, sections were incubated for 3 h with Alexa 488-labeled goat anti-rabbit antibody (1:1000, Invitrogen, Waltham, MA, USA) in PBS, 10% NGS, and 0.1% Triton X-100. DAPI (4′,6-diamidino-2-phenylindol) was used to visualize cell nuclei (1:100 in PBS; 10 min). Sections were washed, transferred onto glass microscope slides, and covered with glass coverslips using Fluoromount G (Southern Biotech, Birmingham, AL, USA).

Confocal images were acquired using a Leica TCS SP8 laser-scanning microscope equipped with a Leica 63× glycerol-immersion objective lens (NA 1.3) (Leica, Bensheim, Germany) according to [40]. All high-resolution images (63× objective lens; 6.8× scan zoom) were acquired at tissue levels ~5 µm below the surface. Microscope settings such as detector gain and amplifier were kept constant during the entire image acquisition. The number and average size of synaptopodin and GluR1 clusters were analyzed in different layers of the dentate gyrus (DG) and the cornu ammonis (CA) 1 region of hippocampus. DG: inner (IML) and outer (OML) molecular layer; CA1: Stratum lacunosum (LA), stratum radiatum (RAD), and stratum oriens (O). For each layer, three visual fields (1024 × 1024 pixels, 27.54 µm × 27.54 µm = 758 µm^2^) were analyzed. Quantitative analysis was performed using the ImageJ software (version 1.52) package (http://rsb.info.nih.gov/ij (accessed on 4 April 2019)). A constant threshold value in the cell free neuropil was set for all analyses. All particles above this threshold with a minimum size of 0.02 µm and a circularity of 0.01–1.00 µm were defined as clusters and counted automatically (“analyze particles” function of ImageJ software). The means of numbers and the average sizes of clusters of the three frames of each layer were calculated [40,41]. The number of clusters was normalized to mm^2^. The values in the respective layers of CA1 and DG were averaged for the (former) light (ZT/CT/DT 02-10) and (former) dark phases (ZT/CT/DT 14-22), respectively. Image acquisition and analysis were performed by an investigator that was blinded to the experimental conditions.

### 2.6. Golgi Staining

To examine the morphology of dendritic spines, an FD-Rapid GolgiStain kit (FD NeuroTechnologies, Columbia, SC, USA) was used according to the manufacturer’s instructions. Briefly, native brains were carefully dissected, washed with cold distilled water, and immersed in a 1:1 premix of solution A and B for 1 day followed by 13 days in solution A and B in darkness. Then, the brains were incubated for 72 h in solution C. Brains were frozen and sectioned on a cryostat (Leica, Bensheim, Germany) into 100 µm coronal sections throughout the rostro-caudal axis of the hippocampus. Sections were mounted onto a drop of solution C on gelatin-coated slides (Marienfeld Superior, Lauda-Königshofen, Germany) and left to dry overnight. Slides were rinsed with cold distilled water followed by impregnation in Golgi staining solution (premix of solution D and solution E with distilled water) for 10 min. Slides were rinsed again with cold distilled water and then dehydrated in ascending alcohol concentrations (50%, 70%, 95%, and 100%), followed by xylol, and then cover-slipped using Entelan. For the analysis, six independent third-order branches of the apical dendrites of CA1 region pyramidal neurons were selected for each mouse. Z-stack images of dendritic spines were acquired using the 100× oil objective in the bright-field mode of a Keyence microscope (Keyence, Osaka, Japan). Spine morphology analysis was performed using SpineJ plugin of ImageJ software. The spine number in each dendrite segment of 50 µm was counted. The spine density was expressed as the number of spines per 50 µm dendrite length. The spine area and spine length of 20 individual spines in each dendrite segment of 50 µm were analyzed. Image acquisition and analysis were performed by an investigator that was blinded to the experimental conditions.

### 2.7. Statistical Analysis

Statistical analysis was performed using GraphPad Prism software 8.0.0 (GraphPad Software, San Diego, CA, USA). Data are presented as the mean ± *SEM*. The Gaussian distribution was tested using the Kolmogorov–Smirnov normality test and visualized by QQ plots. If the data passed the normality tests, differences among groups were analyzed by one-way ANOVA followed by Sidak’s post hoc test for multiple comparison. If data failed normality tests, the Kruskal–Wallis test with Dunn’s multiple comparison test was used. The effects of two parameters, e.g., differences in time curves of corticosterone levels, were analyzed by two-way ANOVA on rank in Origin 2020 (OriginLab Corporation, Northampton, MA, USA). A *p*-value < 0.05 was considered statistically significant.

## 3. Results

### 3.1. Spontaneous Locomotor Activity Is Rhythmic under Acute Constant Darkness and Acute Constant Light

**Figure 1:**
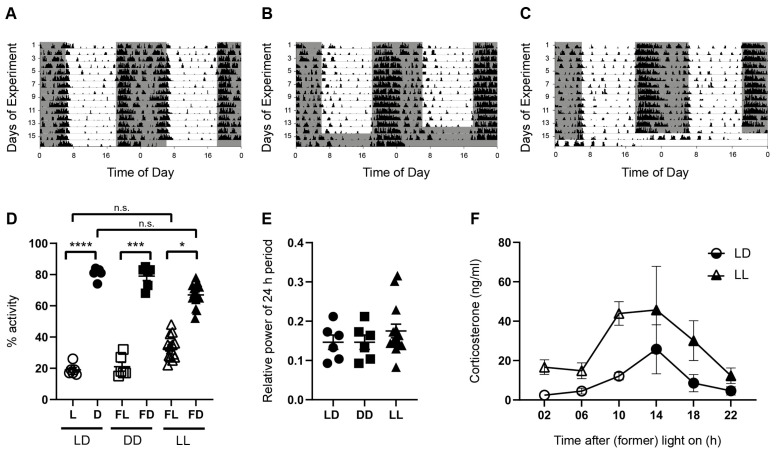
Rhythms in spontaneous locomotor activity and plasma corticosterone levels. Representative double-plotted actograms of spontaneous locomotor activity of mice kept for 14 days in a standard photoperiod of 12 h light (L) and 12 h darkness (D), followed by at least 38 h of (**A**) LD, (**B**) constant darkness (DD), or (**C**) constant light (LL). Black bars indicate activity. Gray boxes indicate periods of darkness. (**D**) Proportion of activity as a percentage of total activity in L or former L (FL) (open symbols), and D or former D (FD) (closed symbols) of mice kept in LD (circles), DD (squares), or LL (triangles). Bars represent the mean ± *SEM* of six mice per group (LD and DD) and 18 mice per group (LL). Kruskal–Wallis test: n.s., not significant; * *p* < 0.05; *** *p* < 0.001; **** *p* < 0.0001. (**E**) Power analysis of the 24 h period in mice kept in LD, DD, and LL. Bars represent the mean ± *SEM* of six mice per group (LD and DD) and 18 mice per group (LL) (Kruskal–Wallis Test). (**F**) Serum corticosterone levels were rhythmic (effect of time) but higher in mice kept in LL than in mice kept in LD (effect of lighting condition). Two-way ANOVA on rank, *p* < 0.0001. Bars represent the mean ± *SEM* of three mice per timepoint.

To show the effect of the lighting conditions on rhythmic spontaneous locomotor activity, mice were kept for 14 days in a standard photoperiod of 12 h light and 12 h darkness (LD), followed by at least 38 h in LD (*n* = 6), in constant darkness (DD, *n* = 6), or in constant light (LL, *n* = 18) (Figure 1). The mice were considered as rhythmic when the percentage of locomotor activity during the light/former light phase was significantly different from the percentage of activity during the dark/former dark phase, and when the relative power of the 24 h period was not affected.

As expected for nocturnal rodents, mice in LD showed a higher proportion of spontaneous locomotor activity during the dark phase (D) as compared to the light phase (L) (*p* < 0.0001) (Figure 1A,D). Mice kept in DD showed a similar pattern of the locomotor activity with high activity during the former dark phase (FD) and low activity during the former light phase (FL) (*p* = 0.0002) (Figure 1B,D), indicating that the circadian system drives rhythmic activity in the absence of rhythmic zeitgeber. Although the pattern of locomotor activity was slightly changed under LL conditions (Figure 1C), locomotor activity was still significantly higher during the former dark phase than during the former light phase (Figure 1D) (*p* = 0.0439). There were no significant differences between the percentage activity in the L (LD) and FL (LL) or between D (LD) and FD (LL) (Figure 1D). Consistently, there was no difference in the relative power of the 24 h period, a measure for rhythmicity, among the different lighting conditions (Figure 1E). Taken together, this indicated that acute LL does not lead to arrhythmicity.

Although serum corticosterone levels of mice kept in LL are still rhythmic (effect of time, *p* < 0.0001), they are significantly higher than in mice kept in LD (effect of lighting condition, *p* < 0.0001) (Figure 1F). This indicates an activation of the endocrine stress axis in response to acute constant light.

### 3.2. Hippocampus-Dependent Spatial Working Memory Is Affected by the Light Phase and by Acute Constant Light

**Figure 2:**
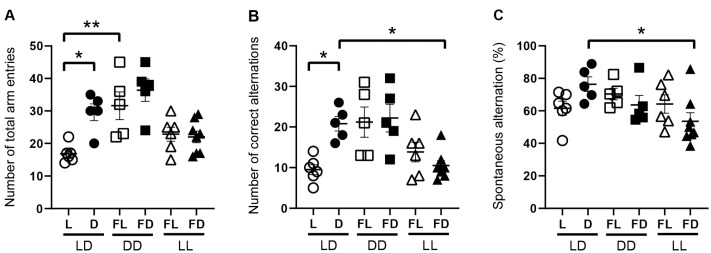
Hippocampus-dependent spatial working memory is affected by light. Exploration activity and correct alternations between arms in the Y-maze of mice kept in a standard photoperiod (LD) of 12 h light (L) and 12 h darkness (D), in constant darkness (DD), or in constant light (LL). The Y-maze test was performed in L or former L (open symbols) and D or former D (closed symbols) of mice kept in LD (circles), DD (squares), or LL (triangles). (**A**) Number of total arm entries reflects exploration activity, (**B**) number of correct alternations reflects the combination of exploration activity and spatial working memory, and (**C**) spontaneous alternation [calculated as the number of correct alternations/(total number of entries −  2)  ×  100%] reflects spatial working memory corrected for exploration activity. Bars represent the mean ± *SEM* of 6–7 mice per group. One-way ANOVA (**A**,**C**), Kruskal–Wallis test (**B**): * *p* < 0.05; ** *p* < 0.01.

To show the effect of light on hippocampus-dependent working memory, mice were kept under LD, acute constant darkness (DD), or acute constant light (LL) and subjected to the Y-maze in L, FL, D, or FD (Figure 2, Videos S1–S6). As performance in the Y-maze is highly related to exploration activity, we first analyzed the number of total arm entries (Figure 2A). In mice kept in LD, the number of total arm entries was significantly higher in the dark phase than in the light phase (*p* = 0.0123) (Figure 2A). In contrast, in mice kept in DD and in mice kept in LL, the number of total arm entries was not different between FL and FD (Figure 2A). This suggests that there is no circadian rhythm in exploratory activity, in contrast to spontaneous locomotor activity (Figure 1). The number of total arm entries was significantly higher in FL of mice kept in DD than in L of mice kept in LD (*p* = 0.0029) (Figure 2A), indicating that, in mice kept in LD, the light phase suppressed exploratory activity. Similarly, the number of correct alternations, which is correlated with explorative behavior, was significantly higher in D than in L of mice kept in LD (*p* = 0.0409), not different between FL and FD of mice kept in DD or LL, and significantly lower in FD of mice kept in LL than in D of mice kept in LD (*p* = 0.0461) (Figure 2B). This suggests that light suppresses exploratory behavior-dependent spatial working memory. Moreover, spontaneous alternations, reflecting spatial working memory corrected by exploration activity, are not different between L and D in mice kept in LD but significantly higher in D of mice kept in LD than in FD of mice kept in LL (*p* = 0.0217) (Figure 2C). This indicates that acute constant light impairs spatial working memory, independent of its effect on explorative activity.

### 3.3. Constant Light Affects Hippocampal Spine Morphology

**Figure 3:**
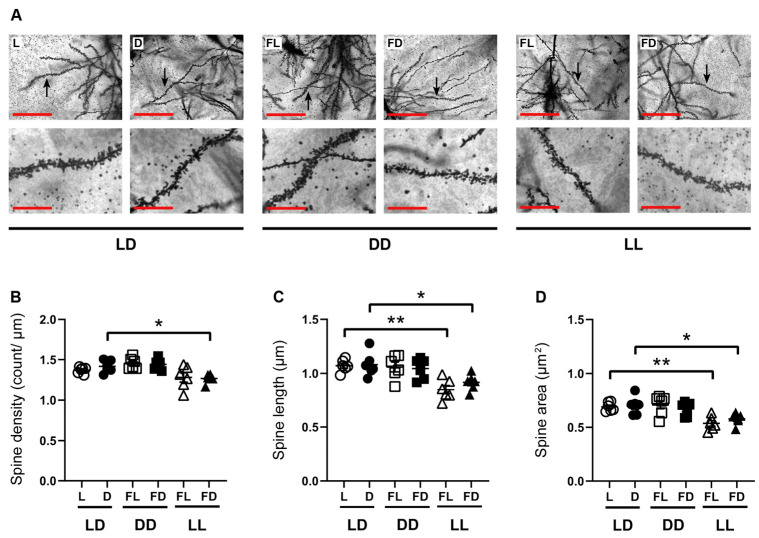
Hippocampal spine morphology is affected by acute constant light. (**A**) Representative microphotographs of Golgi–Cox-stained apical dendritic spines in the dorsal hippocampus of mice kept in a standard photoperiod (LD) of 12 h light (L) and 12 h darkness (D), in constant darkness (DD), or in constant light (LL), sacrificed in L, D, former L (FL), or former D (FD). Scale bar, 50 µm. Higher magnification of the dendrite indicated by the arrow. Scale bar, 12.5 µm. Quantification of (**B**) spine density, (**C**) spine length, and (**D**) spine area of mice kept in LD (circles), DD (squares), or LL (triangles) and sacrificed in L or former L (open symbols) and D or former D (closed symbols). Bars represent the mean ± *SEM* (*n* = 6 hippocampal slides of three mice per group). One-way ANOVA: * *p* < 0.05; ** *p* < 0.01.

Spatial working memory is related to synaptic plasticity of the dorsal hippocampus, while the ventral hippocampus is more strongly associated with emotions and affective behavior. To analyze the effects of light on hippocampal spine morphology of the apical dendrites of pyramidal cells in the CA1 region of the dorsal hippocampus, mice were kept for 14 days in LD, followed by at least 38 h LD (*n* = 6), DD (*n* = 6), or LL (*n* = 6), and sacrificed in L, FL, D, or FD (Figure 3). The spine density (Figure 3B), the spine length (Figure 3C), and the spine area (Figure 3D) were not different between L and D in mice kept in LD, and between FL and FD of mice kept in DD or LL. However, in mice kept in LL and sacrificed in FD, spine density (*p* = 0.0223) (Figure 3B), spine length (*p* = 0.0272) (Figure 3C), and spine area (*p* = 0.0243) (Figure 3D) were reduced compared to mice kept in LD and sacrificed in D. Similarly, in mice kept in LL and sacrificed in FL, the spine length (*p* = 0.0018) (Figure 3C) and spine area (*p* = 0.0042) (Figure 3D) were reduced compared to mice kept in LD and sacrificed in L. Thus, constant light leads to a reduction in the density and size of hippocampal dendritic spines.

### 3.4. Hippocampal Synaptopodin Is Affected by the Light/Dark Phase and Acute Constant Light

**Figure 4:**
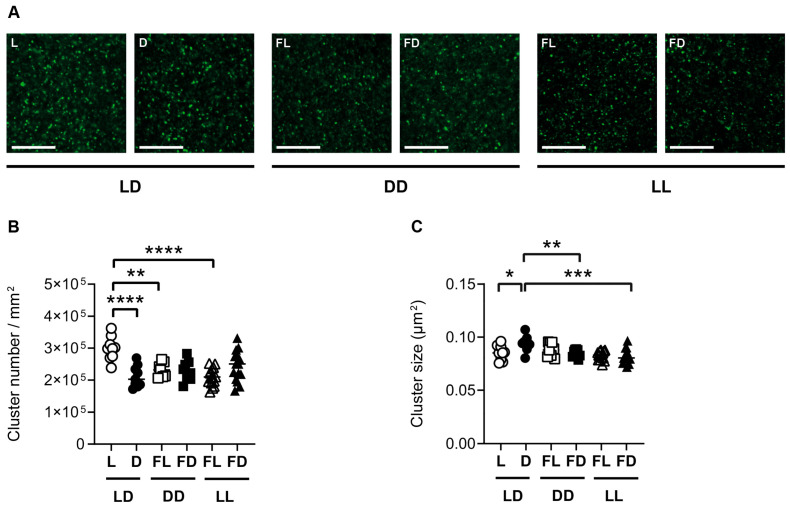
Synaptopodin is affected by the light/dark phase and acute constant light. (**A**) Representative confocal laser microscopic pictures of synaptopodin immunoreactive clusters in the CA1 region of the dorsal hippocampus of mice kept in a standard photoperiod (LD) of 12 h light (L) and 12 h darkness (D), in constant darkness (DD), or in constant light (LL), sacrificed in L, D, former L (FL), or former D (FD). Scale bar, 20 µm. Quantification of (**B**) cluster number and (**C**) cluster size of mice kept in LD (circles), DD (squares), or LL (triangles) and sacrificed in L or FL (open symbols) and D or FD (closed symbols). Bars represent the mean ± *SEM* of *n* = 9 mice per group in LD and DD and *n* = 15 mice per group in LL. One-way ANOVA: * *p* < 0.05; ** *p* < 0.01; *** *p* < 0.001; **** *p* < 0.0001.

In order to investigate the effect of light on hippocampal synaptic plasticity at the molecular level, synaptopodin cluster numbers and sizes in both the CA1 region (Figure 4) and the DG (Figure S1) of the dorsal hippocampus were analyzed in mice kept in 12 h light/12 h darkness (LD, *n* = 18), constant darkness (DD, *n* = 18), or constant light (LL, *n* = 30), and sacrificed in L, FL, D, or FD. In the CA1 region of mice kept in LD, the *number* of synaptopodin clusters (Figure 4B) was higher (*p* < 0.0001), while the *size* of synaptopodin clusters (Figure 4C) was lower (*p* = 0.0169) in L than in D. This indicates that in the dorsal hippocampus of mice kept in LD, the light phase led to an increase in the *number* while the dark phase led to an increase in the *size* of synaptopodin clusters. In contrast, in mice kept in DD, the number and size of synaptopodin clusters were not different between FL and FD in both the CA1 region (Figure 4B,C) and the DG (Figure S1B,C). This indicates that there is no circadian rhythm in synaptopodin cluster dynamics in the dorsal hippocampus, in contrast to spontaneous locomotor activity (Figure 1). Moreover, the number of synaptopodin clusters was lower in FL of mice kept in DD than in L of mice kept in LD in both the CA1 (*p* = 0.002) (Figure 4B) and the DG (*p* = 0.0003) (Figure S1B). This is consistent with an induction in synaptopodin cluster number by light. However, in mice kept in LL and sacrificed in FL, the number of synaptopodin clusters was lower compared to mice kept in LD and sacrificed in L in both the CA1 region (*p* < 0.0001) (Figure 4B) and the DG (*p* < 0.0001) (Figure S1B). Similarly, the size of synaptopodin clusters was lower in mice kept in LL and sacrificed in FD compared to mice kept in LD and sacrificed in D in both the CA1 region (*p* = 0.0001) (Figure 4C) and the DG (*p* < 0.0001) (Figure S1C). This indicates that constant light suppresses the light- and dark-induced increase in synaptopodin cluster number and size, respectively. This is consistent with the suppressive effect of constant light on hippocampus-dependent spatial working memory and spine density.

### 3.5. Hippocampal GluR1 Is Affected by the Light/Dark Phase and Acute Constant Light

**Figure 5:**
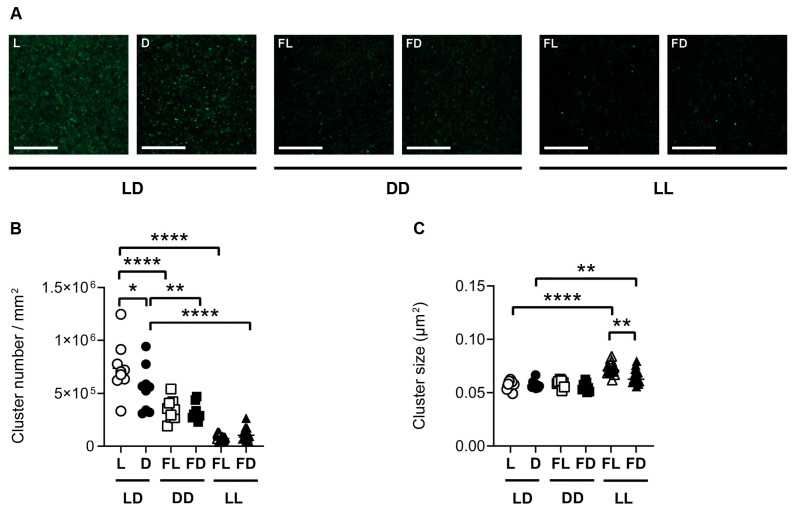
GluR1 is affected by the light/dark phase and acute constant light. (**A**) Representative confocal laser microscopic pictures of GluR1 immunoreactive clusters in the CA1 region of the dorsal hippocampus of mice kept in a standard photoperiod (LD) of 12 h light (L) and 12 h darkness (D), in constant darkness (DD), or in constant light (LL), sacrificed in L, D, former L (FL), or former D (FD). Scale bar, 20 µm. Quantification of (**B**) cluster number and (**C**) cluster size of mice kept for 14 days in LD (circles), DD (squares), or LL (triangles) and sacrificed in L or FL (open symbols) and D or FD (closed symbols). Bars represent the mean ± *SEM* of *n* = 9 mice per group in LD and DD and *n* = 15 mice per group in LL. One-way ANOVA: * *p* < 0.05; ** *p* < 0.01; **** *p* < 0.0001.

Synaptopodin is functionally and structurally linked to AMPA-type glutamate receptors. To investigate the structural association in response to the different lighting conditions, we analyzed GluR1 in parallel sections of those used for the analysis of synaptopodin clusters. Similar to synaptopodin, in the CA1 region and in the DG of mice kept in LD, the *number* of GluR1 clusters (Figure 5B and Figure S2B) was higher in L than in D (CA1: *p* = 0.0255; DG: *p* = 0.0094). In contrast to synaptopodin, the *size* of GluR1 clusters in the CA1 region of the hippocampus and in the DG was not different between L and D in mice kept in LD (Figure 5C and Figure S2C). This indicates that, in the dorsal hippocampus of mice kept in LD, the light phase led to an increase in the *number* of GluR1 clusters while it had no effect on GluR1 cluster *size*. In mice kept in DD, the number and size of GluR1 clusters were not different between FL and FD in both the CA1 region (Figure 5B,C) and the DG (Figure S2B,C). This indicates that there is no circadian rhythm in GluR1 cluster number in the dorsal hippocampus, in contrast to spontaneous locomotor activity (Figure 1). Similar to synaptopodin, the number of GluR1 clusters in the CA1 region of the hippocampus, as well as in the DG, was lower in FL of mice kept in DD (CA1 and DG: *p* < 0.0001) and in FL of mice kept in LL than in L of mice kept in LD (CA1 and DG: *p* < 0.0001) (Figure 5B and Figure S2B). This suggests that constant lighting conditions lead to a downregulation in the *number* of GluR1 in the hippocampus. In contrast to synaptopodin, the *size* of GluR1 clusters was higher in mice kept in FL (CA1: *p* < 0.0001; DG: *p* = 0.0004) and FD (CA1: *p* = 0.0037; DG: *p* = 0.0008) of mice kept in LL than in the respective phase of mice kept in LD (Figure 5C and Figure S2C). This increase in GluR1 cluster size may have compensated for the decrease in cluster number in LL.

## 4. Discussion

Our study showed light/dark differences in hippocampus-dependent spatial working memory and in morphological equivalents of hippocampal excitatory synapses in mice kept under light/dark conditions. These differences were abolished under constant dark conditions although spontaneous locomotor activity was still rhythmic. The findings suggest that light has a greater influence on hippocampal function and the plasticity of excitatory synapses in the hippocampus than rest/locomotor activity. Furthermore, we showed that acute constant light affects hippocampus-dependent spatial working memory and structural plasticity of excitatory synapses and increases corticosterone levels.

### 4.1. Effect of Light/Dark Cycle

In mice kept in LD, the number of correct alternations in the Y-maze, which is dependent on hippocampal plasticity and related to exploratory activity [37], was significantly lower in the light phase than in the dark phase. This is consistent with the suppressive effect of light on activity in nocturnal animals [4], differences in effects of light and dark phase testing on behavioral readouts [42,43], and inhibition of behavioral and cognitive performance in the light phase [44] in mice. In contrast, when the mice were kept in DD to unmask circadian rhythms, there was no difference in the number of correct alternations in the Y-maze, although rhythmic spontaneous locomotor activity persisted. This indicates that the hippocampus-dependent spatial working memory is more strongly driven by light than by circadian rhythms and/or rest/locomotor activity.

It is known from the literature that, in the somatosensory cortex, the density of excitatory synapses is higher during the light phase, while the density of inhibitory synapses is higher during the dark phase [34]. Moreover, under DD, the difference in the density of excitatory synapses was abolished, whereas the differences in the inhibitory synapses persisted, indicating that excitatory synapses are influenced by light, while the inhibitory synapses are driven by the rest/locomotor activity rhythm and/or the circadian clock [34]. Therefore, we focused on the effect of the light/dark conditions on morphological equivalents of excitatory synapses. Indeed, in mice housed in LD, we found a higher *number* of synaptopodin and GluR1 clusters in the light phase than during the dark phase. In mice kept under DD conditions, the differences in the number of synaptopodin and GluR1 clusters were abolished, and the number was reduced in FL of DD compared to L of LD. This is consistent with findings in the somatosensory cortex, indicating that in both the isocortex [34] and the allocortex (this study), the density of excitatory synapses is driven by light rather than rest/locomotor activity rhythm and/or the circadian clock. Since synaptopodin is mainly expressed in mature spines of excitatory synapses [25,26], the higher number of synapotopodin clusters during the light phase might also reflect changes in spine maturation. This is consistent with an increased number of mushroom-shaped spines during the light phase in the somatosensory cortex [45], suggesting that the light phase might also promote spine maturation in the hippocampus.

Furthermore, in mice kept in LD, the *size* of synaptopodin clusters, which is related to synaptic strength [17], was higher during the dark phase than during the light phase. Thus, during the dark phase, an upscaling of synaptic strength may compensate for the reduction in the number of mature spines. Importantly in the dark phase, the larger size of synaptopodin clusters may be linked to a better performance in the Y-maze. In the primary motor and somatosensory cortices, the size of the axon–spine interface was also larger during the dark phase when the mice were awake than during the light phase when the mice were sleeping [46]. However, here, the size of the axon–spine interface increased with enforced activity in the light phase, suggesting an impact of activity on scaling of synaptic strength [46]. Unfortunately, since the study by de Vivo et al. [46] did not include constant dark conditions, it is difficult to distinguish the pure effects of light and rest/activity on scaling of synaptic strength in the primary motor and somatosensory cortices. In our study, in DD, there were no differences in the size of synaptopodin clusters and in the performance in the Y-maze between the former dark phase, when animals showed a higher locomotor activity, and the former light phase, when animals were mainly inactive/resting. This suggests that the light/dark cycle rather than the rhythm in rest/locomotor activity drives rhythmic scaling of synaptic strength (as reflected by changes in synaptopodin) in the hippocampus. In this context, it is interesting to speculate whether light/dark-induced changes in network activity account for the diurnal effects in synaptopodin cluster properties or if other factors, such as hormones, are involved in this process.

According to the literature, under LD conditions, the magnitude of hippocampal LTP in C57Bl/6 mice is generally larger during the dark phase than during the light phase [47]. This is consistent with better spatial learning associated with higher synaptic strength in the dark phase. In C3H mice, which produce melatonin in contrast to C57BL/6 mice, hippocampal LTP is still rhythmic in DD, albeit with a reduced amplitude [47]. There is evidence that the residual rhythm of LTP in C3H mice in DD [47] could be attributed to melatonin [48]. Unfortunately, LTP was not analyzed in C57BL/6 mice in DD [47], the mouse strain used in our study. Synaptopodin contributes to hippocampal LTP [25,49,50] primarily via the Ca^2+^-dependent recruitment of GluR1 to spine synapses [26]. In contrast to the size of synaptopodin clusters, the size of GluR1 cluster was not different between L and D under LD conditions. However, this could be due to the fact that only about 20–30% of spines contain synaptopodin [27]; thus, the effect of light/darkness cannot have such a strong impact on total GluR1. Consistently, there was no difference in the density and length or area of Golgi-stained spines between light and dark phase.

In addition to melatonin, corticosterone, which can be crucially linked to stress responses, seems to be important regulator for hippocampal structural synaptic plasticity [33]. Moreover, stress-induced changes in synaptopodin expression have been reported [51]. However, like spontaneous locomotor activity and body temperature, the rhythm in corticosterone levels is controlled by the circadian clock and, therefore, persists in constant darkness [52,53]. Thus, it is the light phase rather than circadian rhythms that drives the light/dark differences observed in our study. There are several ways that light information can reach the hippocampus. The major input to the hippocampus is through the entorhinal cortex, which projects to both the dentate gyrus and the very distal apical dendrites of pyramidal neurons in the cornu ammonis subfields. The entorhinal cortex, which processes information about space [54] and time, primarily receives input from the visual and association cortices. Moreover, the entorhinal cortex receives input from the post-rhinal area, which integrates spatial and nonspatial visual information and is strongly connected to the amygdala, which provides emotional/affective components. The ipRGCs signal light information via the hypothalamus and brain stem, and via subcortical forebrain regions including the amygdala [7,8]. Neurotransmitters from the brain stem and subcortical forebrain, such as serotonin, norepinephrine, and acetylcholine, are modulators of synaptic plasticity in the hippocampus and, importantly, have a higher basal level during the dark phase compared to the light phase in nocturnal rodents [55], consistent with our hypothesis of the effect of the light/dark phase on synaptic strength. However, we found no apparent differences in the effect of the light/dark phase in the dorsal hippocampus between the CA1 region and the dentate gyrus or among the different layers that could be attributed to a modulation by specific brainstem projections.

### 4.2. Effect of Acute Constant Light

In LL, the plasma levels of corticosterone were increased and the performance in the Y-maze was reduced compared to LD. Moreover, spine density, spine length, and size of hippocampal Golgi-stained spines were reduced in LL compared to LD. This indicates that LL leads to a downscaling in the number and to a change in the morphology of excitatory synapses, thus driving a change in structural plasticity, the central cellular mechanism that underlies memory formation [56]. Spine length and spine area provide information about the maturity and strength of synapses. Mature spines, e.g., mushroom spines, have a larger spine head and contain more glutamate receptors, and they positively correlate with stability and strength of synapses [34,57]. On the other hand, the longer the spine is, the higher the degree of isolation of the spine is from its parent dendrite, which can control the effectiveness of excitatory synapse [58]. This change in structural plasticity is consistent with the suppressive effect of constant light on hippocampus-dependent spatial working memory and with the reduction in density of synaptopodin- and GluR1 clusters in LL. Interestingly, while the size of synaptopodin clusters was reduced, the size of GluR1 was increased in LL. Chronic exposure of mice to LL (for 3–4 weeks) results in elevation and disruption of circadian rhythms in corticosterone plasma levels [13], impairs hippocampal long-term potentiation [59], and disrupts circadian rhythms in locomotor activity [14]. Chronic LL also leads to depressive- and anxiety-like behavior and impaired spatial memory [60]. Similarly, mice kept for 2 weeks in aberrant light conditions of ultrashort days with 3.5 h light/3.5 h darkness (T7 cycle) showed increased corticosterone levels, increased depression-like behavior, and impaired hippocampal LTP [61]. However, it is remarkable that even acute LL used in this study had such significant effects on glucocorticoid levels, spatial memory, and hippocampal structural plasticity. Importantly, locomotor activity and corticosterone levels are still rhythmic in acute LL, indicating that the observed changes were not due to a general disruption of circadian rhythms but more likely to the increase in corticosterone levels. This is consistent with the important role of corticosterone for hippocampal structural synaptic plasticity [33] and with the stress-induced higher levels of glucocorticoids impairing spatial learning [62]. Moreover, treatment with the serotonin-reuptake inhibitor fluoxetine rescues increased corticosterone levels, as well as learning and LTP deficits induced by T7 cycle, supporting the importance of glucocorticoids in the deterioration of hippocampal function/synaptic plasticity due to aberrant light conditions [61]. However, further studies are required to prove the hypothesis that the changes in structural synaptic plasticity under LL are dependent on glucocorticoids. Interestingly, ablation of ipRGCs rescues the spatial learning deficits and the hippocampal LTP decrement induced by the T7 cycle, suggesting that the negative influence of the aberrant light cycles on hippocampus-dependent learning requires ipRGCs.

### 4.3. Limitations and Outlook

In this study, we used only male mice because the female estrous cycle adds a complex additional variable that is known to affect synaptic plasticity and hippocampal functions [63]. However, it is important to include female mice in future studies to investigate the interaction of sex hormones and light on structural and functional synaptic plasticity.

## 5. Conclusions

Our study showed that the light/dark cycle rather than rest/locomotor activity drives diurnal differences in spatial working memory and plasticity of excitatory hippocampal synapses. Moreover, acute constant light has a negative impact on spatial working memory and on the density of hippocampal excitatory synapses. Although studies on nocturnal mice have limited translational value in humans, our study may suggest that the predominant indoor activity during the day with low light intensity, as well as artificial nocturnal light, both of which are increasing in modern society, is detrimental for functional and structural plasticity of the hippocampus.

## Data Availability

The data will be made available by the corresponding author on reasonable request.

## Supplementary Materials

The following supporting information can be downloaded at https://www.mdpi.com/article/10.3390/cells12131758/s1: Figure S1. Synaptopodin clusters in the dentate gyrus (DG) of the dorsal hippocampus are affected by light; Figure S2. GluR1 clusters in the dentate gyrus (DG) of the dorsal hippocampus are affected by light; Video S1. Hippocampus-dependent spatial working memory in Y-maze during light phase of LD; Video S2. Hippocampus-dependent spatial working memory in Y-maze during dark phase of LD; Video S3. Hippocampus-dependent spatial working memory in Y-maze during former light phase of DD; Video S4. Hippocampus-dependent spatial working memory in Y-maze during former dark phase of DD; Video S5. Hippocampus-dependent spatial working memory in Y-maze during former light phase of LL; Video S6. Hippocampus-dependent spatial working memory in Y-maze during former dark phase of LL.

## References

[1] Korf H.-W., von Gall C., Pfaff D.W., Volkow N.D. (2021). Circadian Physiology. Neuroscience in the 21st Century.

[2] Reppert S.M., Weaver D.R. (2002). Coordination of circadian timing in mammals. Nature.

[3] Balsalobre A., Brown S.A., Marcacci L., Tronche F., Kellendonk C., Reichardt H.M., Schütz G., Schibler U. (2000). Resetting of circadian time in peripheral tissues by glucocorticoid signaling. Science.

[4] Mrosovsky N. (1999). Masking: History, definitions, and measurement. Chronobiol. Int..

[5] von Gall C. (2022). The Effects of Light and the Circadian System on Rhythmic Brain Function. Int. J. Mol. Sci..

[6] Hattar S., Liao H.W., Takao M., Berson D.M., Yau K.W. (2002). Melanopsin-containing retinal ganglion cells: Architecture, projections, and intrinsic photosensitivity. Science.

[7] Provencio I., Rodriguez I.R., Jiang G., Hayes W.P., Moreira E.F., Rollag M.D. (2000). A novel human opsin in the inner retina. J. Neurosci..

[8] Hattar S., Kumar M., Park A., Tong P., Tung J., Yau K.-W., Berson D.M. (2006). Central projections of melanopsin-expressing retinal ganglion cells in the mouse. J. Comp. Neurol..

[9] Rumanova V.S., Okuliarova M., Zeman M. (2020). Differential Effects of Constant Light and Dim Light at Night on the Circadian Control of Metabolism and Behavior. Int. J. Mol. Sci..

[10] Walker W.H., Walton J.C., DeVries A.C., Nelson R.J. (2020). Circadian rhythm disruption and mental health. Transl. Psychiatry.

[11] Chinoy E.D., Duffy J.F., Czeisler C.A. (2018). Unrestricted evening use of light-emitting tablet computers delays self-selected bedtime and disrupts circadian timing and alertness. Physiol. Rep..

[12] González M.M.C. (2018). Dim Light at Night and Constant Darkness: Two Frequently Used Lighting Conditions That Jeopardize the Health and Well-being of Laboratory Rodents. Front. Neurol..

[13] Claustrat B., Valatx J.L., Harthe C., Brun J. (2008). Effect of constant light on prolactin and corticosterone rhythms evaluated using a noninvasive urine sampling protocol in the rat. Horm. Metab. Res..

[14] Mrosovsky N. (2003). Aschoff’s rule in retinally degenerate mice. J. Comp. Physiol. A Neuroethol. Sens. Neural Behav. Physiol..

[15] Raven F., Van der Zee E.A., Meerlo P., Havekes R. (2018). The role of sleep in regulating structural plasticity and synaptic strength: Implications for memory and cognitive function. Sleep Med. Rev..

[16] Yuste R., Denk W. (1995). Dendritic spines as basic functional units of neuronal integration. Nature.

[17] Vlachos A., Ikenberg B., Lenz M., Becker D., Reifenberg K., Bas-Orth C., Deller T. (2013). Synaptopodin regulates denervation-induced homeostatic synaptic plasticity. Proc. Natl. Acad. Sci. USA.

[18] Vlachos A., Becker D., Jedlicka P., Winkels R., Roeper J., Deller T. (2012). Entorhinal denervation induces homeostatic synaptic scaling of excitatory postsynapses of dentate granule cells in mouse organotypic slice cultures. PLoS ONE.

[19] Turrigiano G. (2012). Homeostatic synaptic plasticity: Local and global mechanisms for stabilizing neuronal function. Cold Spring Harb. Perspect. Biol..

[20] O’Brien R.J., Kamboj S., Ehlers M.D., Rosen K.R., Fischbach G.D., Huganir R.L. (1998). Activity-dependent modulation of synaptic AMPA receptor accumulation. Neuron.

[21] Sutton M.A., Ito H.T., Cressy P., Kempf C., Woo J.C., Schuman E.M. (2006). Miniature neurotransmission stabilizes synaptic function via tonic suppression of local dendritic protein synthesis. Cell.

[22] Pozo K., Goda Y. (2010). Unraveling mechanisms of homeostatic synaptic plasticity. Neuron.

[23] Kopec C.D., Real E., Kessels H.W., Malinow R. (2007). GluR1 links structural and functional plasticity at excitatory synapses. J. Neurosci. Off. J. Soc. Neurosci..

[24] Mundel P. (1997). Synaptopodin: An Actin-associated Protein in Telencephalic Dendrites and Renal Podocytes. J. Cell Biol..

[25] Deller T., Korte M., Chabanis S., Drakew A., Schwegler H., Stefani G.G., Zuniga A., Schwarz K., Bonhoeffer T., Zeller R. (2003). Synaptopodin-deficient mice lack a spine apparatus and show deficits in synaptic plasticity. Proc. Natl. Acad. Sci. USA.

[26] Vlachos A., Korkotian E., Schonfeld E., Copanaki E., Deller T., Segal M. (2009). Synaptopodin regulates plasticity of dendritic spines in hippocampal neurons. J. Neurosci..

[27] Bas Orth C., Vlachos A., Del Turco D., Burbach G.J., Haas C.A., Mundel P., Feng G., Frotscher M., Deller T. (2005). Lamina-specific distribution of Synaptopodin, an actin-associated molecule essential for the spine apparatus, in identified principal cell dendrites of the mouse hippocampus. J. Comp. Neurol..

[28] Deller T. (2000). Actin-associated protein Synaptopodin. J. Comp. Neurol..

[29] Deller T. (2000). Potential Role of Synaptopodin in Spine Motility by Coupling Actin to the Spine Apparatus. Hippocampus.

[30] Yap K., Drakew A., Smilovic D., Rietsche M., Paul M.H., Vuksic M., Del Turco D., Deller T. (2020). The actin-modulating protein synaptopodin mediates long-term survival of dendritic spines. eLife.

[31] Matsuzaki M., Ellis-Davies G.C., Nemoto T., Miyashita Y., Iino M., Kasai H. (2001). Dendritic spine geometry is critical for AMPA receptor expression in hippocampal CA1 pyramidal neurons. Nat. Neurosci..

[32] Matsuzaki M., Honkura N., Ellis-Davies G.C., Kasai H. (2004). Structural basis of long-term potentiation in single dendritic spines. Nature.

[33] Ikeda M., Hojo Y., Komatsuzaki Y., Okamoto M., Kato A., Takeda T., Kawato S. (2015). Hippocampal spine changes across the sleep-wake cycle: Corticosterone and kinases. J. Endocrinol..

[34] Jasinska M., Grzegorczyk A., Woznicka O., Jasek E., Kossut M., Barbacka-Surowiak G., Litwin J.A., Pyza E. (2015). Circadian rhythmicity of synapses in mouse somatosensory cortex. Eur. J. Neurosci..

[35] Debski K.J., Ceglia N., Ghestem A., Ivanov A.I., Brancati G.E., Bröer S., Bot A.M., Müller J.A., Schoch S., Becker A. (2020). The circadian hippocampus and its reprogramming in epilepsy: Impact for chronotherapeutics. bioRxiv.

[36] Kilkenny C., Browne W.J., Cuthi I., Emerson M., Altman D.G. (2012). Improving bioscience research reporting: The ARRIVE guidelines for reporting animal research. Vet. Clin. Pathol..

[37] Lalonde R. (2002). The neurobiological basis of spontaneous alternation. Neurosci. Biobehav. Rev..

[38] Naert A., Gantois I., Laeremans A., Vreysen S., Van den Bergh G., Arckens L., Callaerts-Vegh Z., D’Hooge R. (2013). Behavioural alterations relevant to developmental brain disorders in mice with neonatally induced ventral hippocampal lesions. Brain Res. Bull..

[39] Franklin K.B.J. (2008). The Mouse Brain in Stereotaxic Coordinates/Keith B.J. Franklin, George Paxinos.

[40] Strehl A., Lenz M., Itsekson-Hayosh Z., Becker D., Chapman J., Deller T., Maggio N., Vlachos A. (2014). Systemic inflammation is associated with a reduction in Synaptopodin expression in the mouse hippocampus. Exp. Neurol..

[41] Lenz M., Ben Shimon M., Deller T., Vlachos A., Maggio N. (2017). Pilocarpine-Induced Status Epilepticus Is Associated with Changes in the Actin-Modulating Protein Synaptopodin and Alterations in Long-Term Potentiation in the Mouse Hippocampus. Neural Plast..

[42] Richetto J., Polesel M., Weber-Stadlbauer U. (2019). Effects of light and dark phase testing on the investigation of behavioural paradigms in mice: Relevance for behavioural neuroscience. Pharmacol. Biochem. Behav..

[43] Tsao C.H., Flint J., Huang G.J. (2022). Influence of diurnal phase on behavioral tests of sensorimotor performance, anxiety, learning and memory in mice. Sci. Rep..

[44] Roedel A., Storch C., Holsboer F., Ohl F. (2006). Effects of light or dark phase testing on behavioural and cognitive performance in DBA mice. Lab. Anim..

[45] Jasinska M., Jasek-Gajda E., Woznicka O., Lis G.J., Pyza E., Litwin J.A. (2019). Circadian clock regulates the shape and content of dendritic spines in mouse barrel cortex. PLoS ONE.

[46] de Vivo L., Bellesi M., Marshall W., Bushong E.A., Ellisman M.H., Tononi G., Cirelli C. (2017). Ultrastructural evidence for synaptic scaling across the wake/sleep cycle. Science.

[47] Chaudhury D., Wang L.M., Colwell C.S. (2005). Circadian regulation of hippocampal long-term potentiation. J. Biol. Rhythm..

[48] Wang L.M., Suthana N.A., Chaudhury D., Weaver D.R., Colwell C.S. (2005). Melatonin inhibits hippocampal long-term potentiation. Eur. J. Neurosci..

[49] Fukazawa Y. (2003). Hippocampal LTP Is Accompanied by Enhanced F-Actin Content within the Dendritic Spine that Is Essential for Late LTP Maintenance In Vivo. Neuron.

[50] Yamazaki M., Matsuo R., Fukazawa Y., Ozawa F., Inokuchi K. (2001). Regulated expression of an actin-associated protein, synaptopodin, during long-term potentiation. J. Neurochem..

[51] Vlachos A., Maggio N., Segal M. (2008). Lack of correlation between synaptopodin expression and the ability to induce LTP in the rat dorsal and ventral hippocampus. Hippocampus.

[52] Filipski E., King V.M., Li X., Granda T.G., Mormont M.C., Liu X., Claustrat B., Hastings M.H., Lévi F. (2002). Host circadian clock as a control point in tumor progression. J. Natl. Cancer Inst..

[53] Tsinkalovsky O., Filipski E., Rosenlund B., Sothern R., Eiken H., Wu M., Claustrat B., Bayer J., Lévi F., Laerum O. (2006). Circadian expression of clock genes in purified hematopoietic stem cells is developmentally regulated in mouse bone marrow. Exp. Hematol..

[54] Schmidt-Hieber C., Häusser M. (2013). Cellular mechanisms of spatial navigation in the medial entorhinal cortex. Nat. Neurosci..

[55] Kalén P., Rosegren E., Lindvall O., Björklund A. (1989). Hippocampal Noradrenaline and Serotonin Release over 24 Hours as Measured by the Dialysis Technique in Freely Moving Rats: Correlation to Behavioural Activity State, Effect of Handling and Tail-Pinch. Eur. J. Neurosci..

[56] Kasai H., Fukuda M., Watanabe S., Hayashi-Takagi A., Noguchi J. (2010). Structural dynamics of dendritic spines in memory and cognition. Trends Neurosci..

[57] Jasinska M., Woznicka O., Jasek-Gajda E., Lis G.J., Pyza E., Litwin J.A. (2020). Circadian Changes of Dendritic Spine Geometry in Mouse Barrel Cortex. Front. Neurosci..

[58] Araya R., Vogels T.P., Yuste R. (2014). Activity-dependent dendritic spine neck changes are correlated with synaptic strength. Proc. Natl. Acad. Sci. USA.

[59] Chai A.P., Ma W.P., Wang L.P., Cao J., Xu L., Yang Y.X., Mao R.R. (2015). Chronic constant light-induced hippocampal late-phase long-term potentiation impairment in vitro is attenuated by antagonist of D1/D5 receptors. Brain Res..

[60] Zhou Y., Zhang H.K., Liu F., Lei G., Liu P., Jiao T., Dang Y.H. (2018). Altered Light Conditions Contribute to Abnormalities in Emotion and Cognition through HINT1 Dysfunction in C57BL/6 Mice. Front. Behav. Neurosci..

[61] LeGates T.A., Altimus C.M., Wang H., Lee H.K., Yang S., Zhao H., Kirkwood A., Weber E.T., Hattar S. (2012). Aberrant light directly impairs mood and learning through melanopsin-expressing neurons. Nature.

[62] Krugers H.J., Douma B.R., Andringa G., Bohus B., Korf J., Luiten P.G. (1997). Exposure to chronic psychosocial stress and corticosterone in the rat: Effects on spatial discrimination learning and hippocampal protein kinase Cgamma immunoreactivity. Hippocampus.

[63] Babayan A.H., Kramár E.A. (2013). Rapid effects of oestrogen on synaptic plasticity: Interactions with actin and its signalling proteins. J. Neuroendocr..

