# Supplementary material for: Effects of the Light/Dark Phase and Constant Light on Spatial Working Memory and Spine Plasticity in the Mouse Hippocampus
Source: Cells. 2023 Jun 30;12(13):1758. doi: 10.3390/cells12131758 (PMC10340644; doi:10.3390/cells12131758)
Supplement: Supplementary file 1 [file cells-12-01758-s001.zip › Figures S1 and S2.pdf]

cells

MDPI

Supplementary Materials:

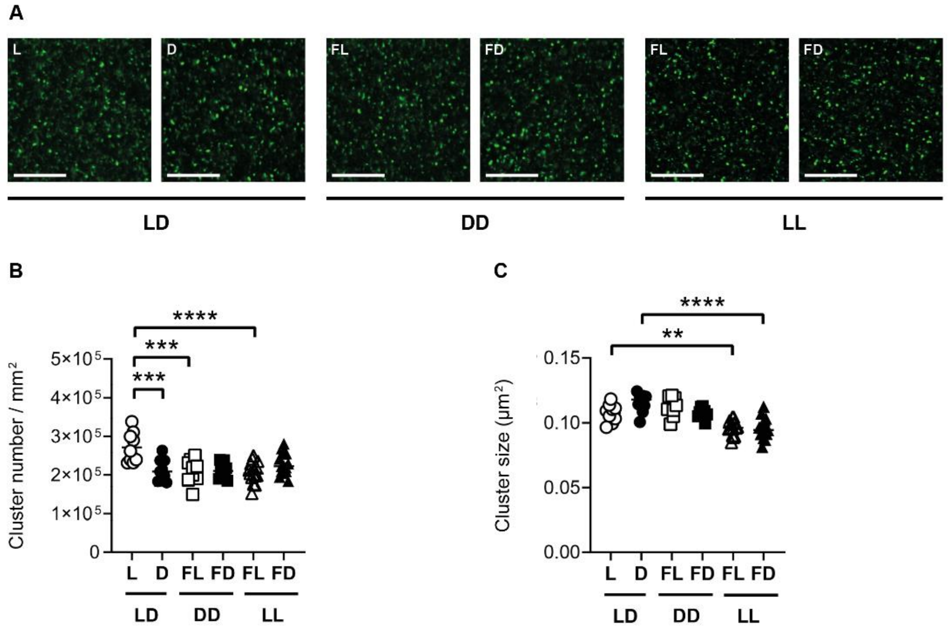

Figure S1 Synaptopodin clusters in the dentate gyrus (DG) of the dorsal hippocampus are affected by light. A) Representative confocal laser microscopic pictures of synaptopodin immunoreactive clusters in the DG of the dorsal hippocampus of mice in a standard photoperiod (LD) of 12 h light (L) and 12 h darkness (D), in constant darkness (DD) or constant light (LL) and sacrificed in L, former L (FL), D, or former D (FD). Scale bar, 20 µm. Quantification of B) cluster size, and C) cluster size of mice kept in LD (circles), DD (squares), or LL (triangles) and sacrificed in L or FL (open symbols) and D or FD (closed symbols). Bars represent mean ±SEM of n=9 mice per group in LD and DD and n=15 mice per group in LL. One-way ANOVA, **: $p < 0.01$, ***: $p < 0.001$, ****: $p < 0.0001$.

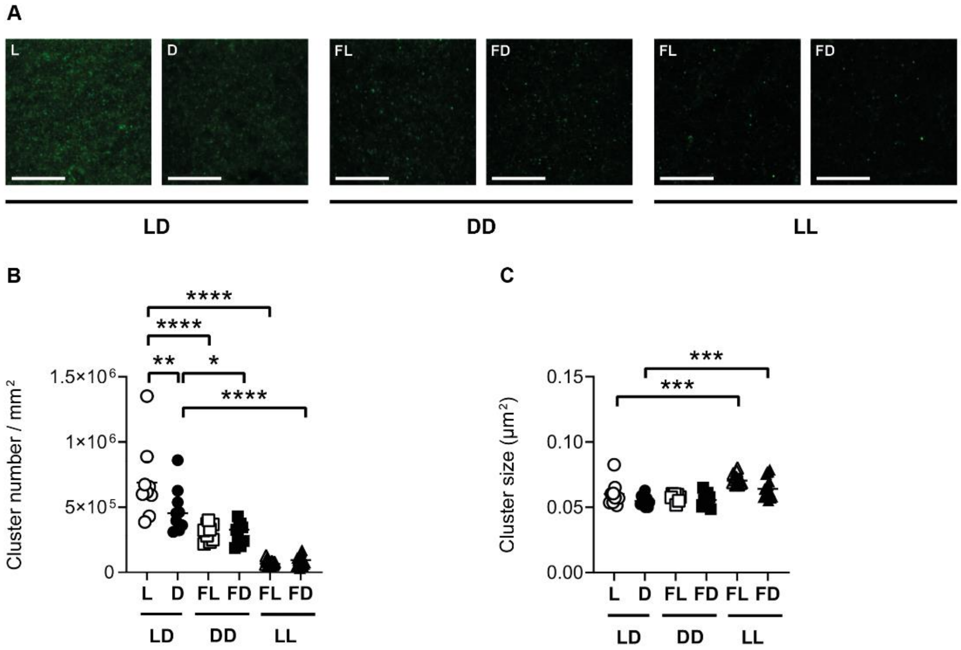

Figure S2 GluR1 clusters in the dentate gyrus (DG) of the dorsal hippocampus are affected by light. A) Representative confocal laser microscopic pictures of GluR1 immunoreactive clusters in the DG of the dorsal hippocampus of mice kept in a standard photoperiod (LD) of 12 h light (L) and 12 h darkness (D), in constant darkness (DD), or constant light (LL) and sacrificed in L, former L (FL), D, or former D (FD). Scale bar, 20 μm. Quantification of B) cluster number, and C) cluster size of mice kept in LD (circles), DD (squares), or LL (triangles) and sacrificed in L or FL (open symbols) and D or FD (closed symbols). Bars represent mean ±SEM of n=9 mice per group in LD and DD and n=15 mice per group in LL. One-way ANOVA, *: $p < 0.05$, **: $p < 0.01$, ***: $p < 0.001$, ****: $p < 0.0001$.
